# Supplementary material for: Components of an ESCRT-independent nuclear envelope assembly pathway
Source: bioRxiv. 2026 Feb 3:2026.02.01.703137. Preprint. [Version 1] doi: 10.64898/2026.02.01.703137 (PMC12889549; doi:10.64898/2026.02.01.703137)
Supplement: Supplement 4 [file NIHPP2026.02.01.703137v1-supplement-4.pdf]

bioRxiv preprint doi: https://doi.org/10.64898/2026.02.01.703137; this version posted February 3, 2026. The copyright holder for this preprint (which was not certified by peer review) is the author/funder, who has granted bioRxiv a license to display the preprint in perpetuity. It is made available under aCC-BY 4.0 International license.

Figure S1

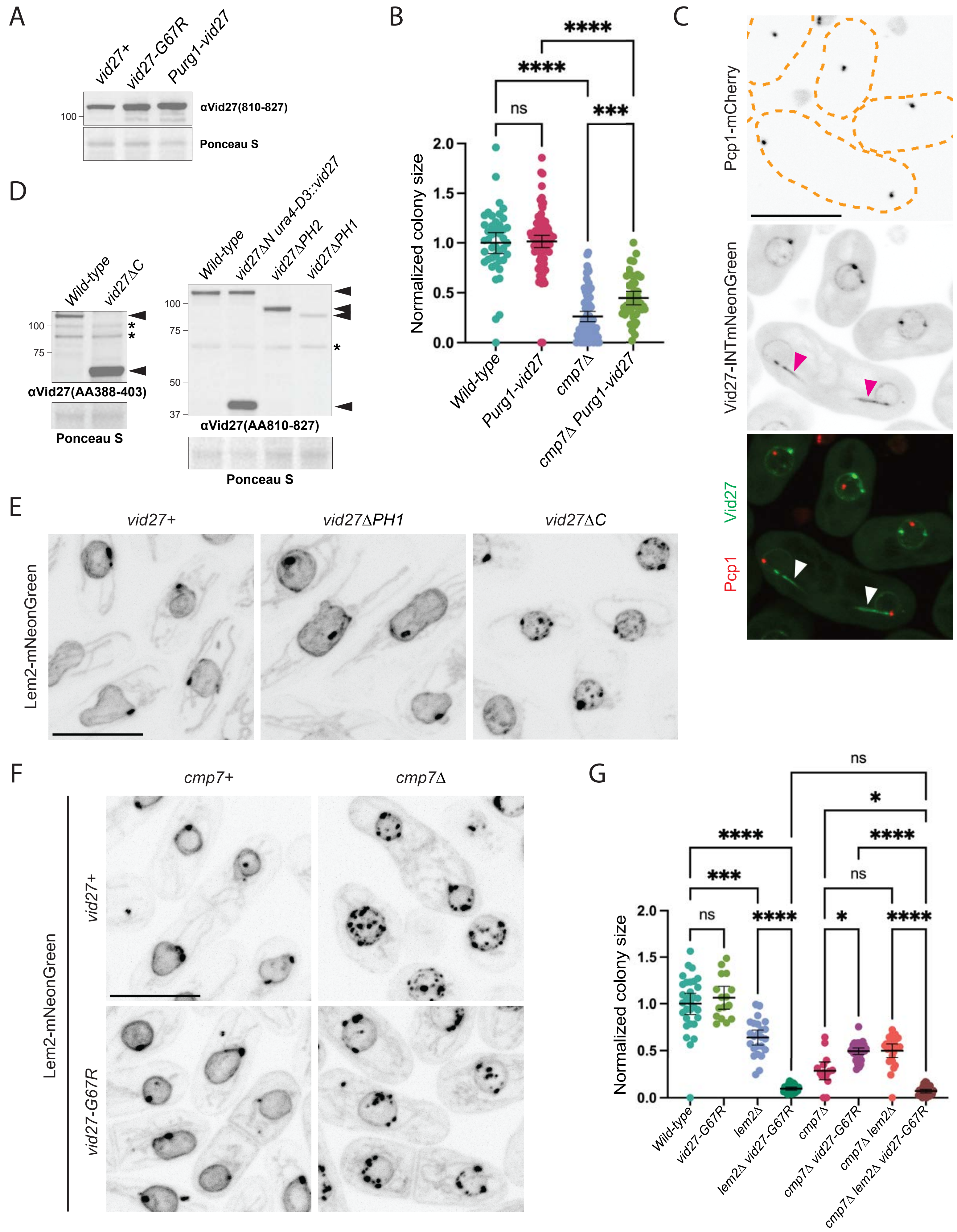

bioRxiv preprint doi: https://doi.org/10.64898/2026.02.01.703137; this version posted February 3, 2026. The copyright holder for this preprint (which was not certified by peer review) is the author/funder, who has granted bioRxiv a license to display the preprint in perpetuity. It is made available under aCC-BY 4.0 International license.

Figure S2

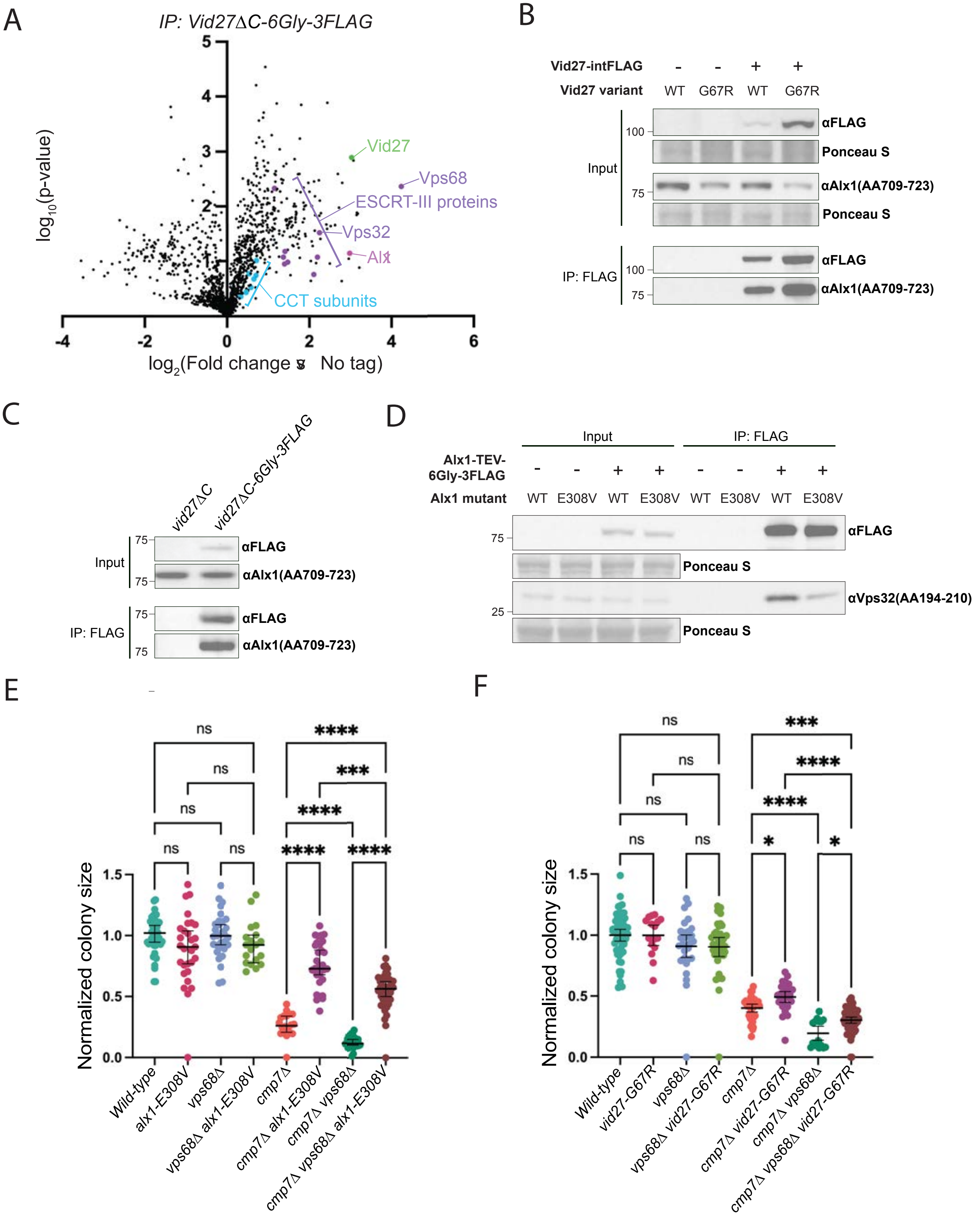

bioRxiv preprint doi: https://doi.org/10.64898/2026.02.01.703137; this version posted February 3, 2026. The copyright holder for this preprint (which was not certified by peer review) is the author/funder, who has granted bioRxiv a license to display the preprint in perpetuity. It is made available under aCC-BY 4.0 International license.

Figure S3

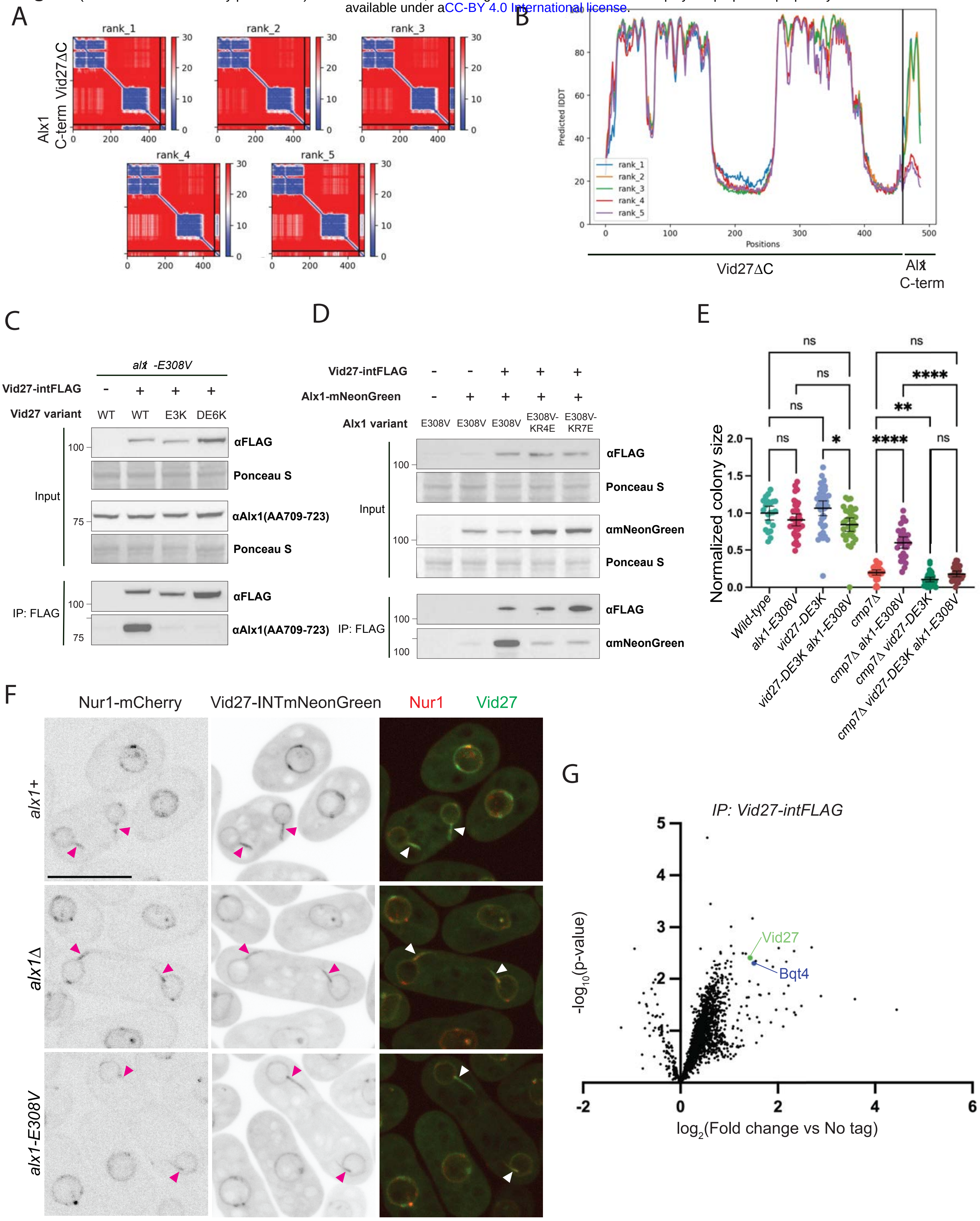

bioRxiv preprint doi: https://doi.org/10.64898/2026.02.01.703137; this version posted February 3, 2026. The copyright holder for this preprint (which was not certified by peer review) is the author/funder, who has granted bioRxiv a license to display the preprint in perpetuity. It is made available under aCC-BY 4.0 International license.

Figure S1

bioRxiv preprint doi: https://doi.org/10.64898/2026.02.01.703137; this version posted February 3, 2026. The copyright holder for this preprint (which was not certified by peer review) is the author/funder, who has granted bioRxiv a license to display the preprint in perpetuity. It is made available under aCC-BY 4.0 International license.

Vid27 promotes NE assembly independent of any effect on Lem2 clustering. (A) As assessed by Western blot, Vid27-G67R levels are comparable to moderate overexpression of Vid27 **(B)** Moderate overexpression of *vid27* suppresses the growth defect of *cmp7Δ* cells. Normalized colony size after ascus dissection. Means ± 95% CI are shown. ****P≤0.0001; ***P≤0.001; ns, not significant; Brown–Forsythe and Welch ANOVA with Dunnett's multiple comparison test. **(C)** Vid27 clusters do not localize to SPBs. Shown are representative images of cells expressing Vid27-INTmNG and Pcp1-mCherry. Arrowheads, Vid27 at mitotic tails; Scale bar, 10 µm. **(D)** Steady-state levels of indicated Vid27 truncations. A covering copy of *VID27* (*ura4-D3::vid27*) was used to generate a viable strain expressing Vid27ΔN. Note that an antibody to a different Vid27 peptide was used for Vid27ΔC since this truncation lacks the peptide recognized by our primary Vid27 antibody. Asterisks, non-specific bands. **(E)** Lem2 forms clusters in *vid27ΔC* cells but not *vid27ΔPH1* cells. Shown are representative images of cells expressing Lem2-mNeonGreen in the indicated genetic backgrounds. Scale bar, 10 µm. **(F)** *cmp7Δ vid27-G67R* cells have Lem2 clusters. Shown are representative images of cells expressing Lem2-mNeonGreen in *cmp7Δ* and *cmp7Δ vid27-G67R* cells. Scale bar, 10 µm. **(G)** *vid27-G67R* has a negative genetic interaction with *lem2Δ.* Normalized colony size after ascus dissection. Means ± 95% CI are shown. ****P≤0.0001; ***P≤0.001; *P≤0.05; ns, not significant; Brown–Forsythe and Welch ANOVA with Dunnett's multiple comparison test.

Figure S2

The N-terminal half of Vid27 interacts with Alx1 as part of an ESCRT-independent NE assembly pathway. (A) IP MS suggests that Vid27ΔC interacts with Alx1. Volcano plots show TMT-based quantitative MS of Vid27ΔC-6Gly-3FLAG IPs relative to untagged control. Statistical comparisons of 3 replicates were made using a t-test. **(B)** Western blot of Vid27 and Vid27-G67R co-IP of Alx1. **(C)** Western blot of Vid27ΔC co-IP of Alx1 **(D)** Reduced interaction between Alx1-E308V and ESCRT-III (Vps32). Western blot for Vps32 after Alx1 and Alx-E308V IP. **(E and F)** *alx1-E308V* and *vid27-G67R* can suppress growth defects of *cmp7Δ* independent of Vps68. Normalized colony size after ascus dissection. Means ± 95% CI are shown. ****P≤0.0001; ***P≤0.001; *P≤0.05; ns, not significant; Brown–Forsythe and Welch ANOVA with Dunnett's multiple comparison test. Note: Although Vps68 is not required for suppression of *cmp7Δ* by *alx1-E308V* or *vid27-G67R*, there is a negative genetic interaction between *cmp7Δ* and *vps68Δ*, raising the possibility that Vps68 could have some function in parallel to Cmp7.

Figure S3

Vid27 and Alx1 form a complex. (A and B) AlphaFold2 confidence metrics for Alx1-Vid27 predicted interaction. **(C and D)** Mutations at the predicted Alx1-Vid27 binding interface disrupt the Alx1-E308V interaction with Vid27. **(C)** IP of Vid27 or Vid27 binding surface mutants assessed for co-IP of Alx1-E308V. **(D)** IP of Vid27 assessed for co-IP of Alx1-E308V or Alx1-E308V binding surface mutants. **(E)** *alx1-E308V* loses ability to suppress the growth defects of *cmp7Δ* cells if the Alx1-Vid27 interaction is disrupted**.** Normalized colony size after ascus dissection. Means ± 95% CI are shown. *P≤0.05; **P≤0.01; ****P≤0.0001; ns, not significant; Brown–Forsythe and Welch ANOVA with Dunnett's multiple comparison test**.** **(F)** Vid27 localization is not affected in *alx1Δ* or *alx1-E308V* strains. Shown are representative images of WT, *alx1Δ,* and *alx1-E308V* cells expressing Vid27-INTmNeonGreen. Scale bar, 10 µm. **(G)** IP MS indicates that Vid27 interacts with Bqt4. Volcano plots show TMT-based quantitative MS of Vid27-intFLAG IPs relative to untagged control. Statistical comparisons of 3 replicates we made using a t test.
